# Draft Genome Sequence of an Extensively Drug-Resistant *Acinetobacter baumannii* Indigo-Pigmented Strain

**DOI:** 10.1128/genomeA.01146-14

**Published:** 2014-11-13

**Authors:** German Traglia, Elisabet Vilacoba, Marisa Almuzara, Leticia Diana, Andres Iriarte, Daniela Centrón, María Soledad Ramírez

**Affiliations:** a Instituto de Microbiología y Parasitología Médica (IMPaM, UBA-CONICET), Facultad de Medicina, Universidad de Buenos Aires, Buenos Aires, Argentina; b Laboratorio de Bacteriología, Hospital Interzonal de Agudos Eva Perón, San Martín Buenos Aires, Buenos Aires, Argentina; c Área Bacteriología, Departamento de Microbiología, Facultad de Veterinaria, UdelaR, Montevideo, Uruguay; d Departamento de Bioquímica y Genómica Microbianas and Departamento Genómica, IIBCE, Montevideo, Uruguay; e Departamento de Desarrollo Biotecnológico, Instituto de Higiene, Facultad de Medicina, UdelaR, Montevideo, Uruguay; f Department of Biological Science, California State University, Fullerton, Fullerton, California, USA

## Abstract

Last year in 2013, we reported an outbreak due to indigo-pigmented *Acinetobacter baumannii* strains in a hospital from Buenos Aires, Argentina. Here, we present the draft genome sequence of one of the strains (*A. baumannii* A33405) involved in the outbreak. This isolate was categorized as extensively drug-resistant (XDR) and harbors different genetic elements associated with horizontal genetic transfer and multiple antibiotic resistances.

## GENOME ANNOUNCEMENT

*Acinetobacter baumannii* has been recognized as one of the major causes of hospital-acquired infections (1, 2). Its ability to develop extreme drug resistance and survive for long periods on inanimate surfaces makes *A. baumannii* one of the most important enemies in intensive care units, being the cause of many outbreaks (1, 3). In the year 2013, we reported an outbreak due to indigo-pigmented *A. baumannii* strains, which are not common in clinical settings (4). Molecular studies revealed that all the isolates belonging to the clonal complex 113^B^ (CC113^B^/CC79^P^) and were extensively drug resistant. They possessed the transposon Tn*2006*, class 2 integrons, AbaR-type islands, IS*125*, IS*26*, *strA*, *strB*, *florR*, and the small recombinase IS*CR2* associated with the *sul2* gene, preceded by IS*Aba1* (4). To date, there are no whole-genome sequences from indigo-pigmented *A. baumannii* strains available in GenBank/EMBL/DDBJ. Here, we report the draft genome sequence of an indigo-pigmented *A. baumannii* strain, A33405, which was isolated from a 65-year-old male patient from a coronary care unit (4).

Using pan-PCR as a molecular technique, this strain was classified as belonging to the CC113^B^/CC79^P^ clonal complex, which was shown to be prevalent in clinical *A. baumannii* isolates from our country (5).

A draft sequence for *A. baumannii* A33405 was developed using Illumina MiSeq at the Argentinian Consortium of Genomic Technology (ACGT). A total of 1,615,755 high-quality paired-end reads were produced, with an average insertion size of 327 reads. *De novo* assembly was performed with the SPAdes assembler version 3.1.0 (6), using a preassembly approach with Velvet (7). Of the generated reads, 99.5% that showed an average length of 231 reads were mapped, resulting in a mean coverage of 31× (sequence depth). The assembled contigs sum 3,892,826 base pairs, with an *N*_50_ contig size of 91,003 (max length, 253,127), and have a G+C content of 39.07%. Open reading frames were predicted and annotated using the RAST server, which identified 3,741 possible open reading frames (ORFs) and 71 copies of rRNA operons (3). Using the tRNAscan-SE, a total of 62 tRNA genes were identified (8).

In order to confirm the result obtained by pan-PCR, *in silico* multilocus sequence typing (MLST) was carried out according to the scheme of Bartual et al. (9) by searching and comparing the internal regions of *gltA, gyrB, gdhB, recA, cpn60, gpi*, and *rpoD* (http://pubmlst.org/abaumannii/). The *in silico* MLST results using the concatenated allele sequences confirm the pan-PCR result, showing that A33405 belongs to sequence type 227 (ST227)/CC113.

The possibility of getting the complete genome sequence of strain A33405, which is not only the first whole-genome sequence of a CC113^B^/CC79^P^ strain but also the first whole-genome sequence of an indigo-pigmented *A. baumannii* strain, is allowing us to get a more detailed analysis and study novel features in *A. baumannii*. Also, it will allow us to perform whole-genome sequence comparisons and phylogenetic analyses, thus expanding our understanding of this global pathogen and its characteristics in different parts of the world. The characterization of the resistance determinants and the virulence factors present in *A. baumannii* strain A33405 will be included in a future publication.

### Nucleotide sequence accession numbers.

This whole-genome shotgun project has been deposited at DDBJ/EMBL/GenBank under the accession no. JPXZ00000000. The version described in this paper is version JPXZ01000000.
